# Learning and Treatment of Anaphylaxis by Laypeople: A Simulation Study Using Pupilar Technology

**DOI:** 10.1155/2017/9837508

**Published:** 2017-07-05

**Authors:** Felipe Fernandez-Mendez, Nieves Maria Saez-Gallego, Roberto Barcala-Furelos, Cristian Abelairas-Gomez, Alexis Padron-Cabo, Alexandra Perez-Ferreiros, Carlos Garcia-Magan, Jose Moure-Gonzalez, Onofre Contreras-Jordan, Antonio Rodriguez-Nuñez

**Affiliations:** ^1^University College of Nursing, University of Vigo, Pontevedra, Spain; ^2^CLINURSID Research Group, Psychiatry, Radiology and Public Health Department, University of Santiago de Compostela, Santiago de Compostela, Spain; ^3^Faculty of Education and Sport Sciences, REMOSS Network Research, University of Vigo, Pontevedra, Spain; ^4^Faculty of Education, University of Castilla la Mancha, Toledo, Spain; ^5^Faculty of Educational Sciences, University of Santiago de Compostela, Santiago de Compostela, Spain; ^6^Pediatric Area, Pediatric Allergy Unit, University of Santiago de Compostela, Santiago de Compostela, Spain; ^7^Faculty of Education, University of Castilla la Mancha, Albacete, Spain; ^8^Pediatric Area, Pediatric Emergency and Critical Care Division, University of Santiago de Compostela, Santiago de Compostela, Spain

## Abstract

An anaphylactic shock is a time-critical emergency situation. The decision-making during emergencies is an important responsibility but difficult to study. Eye-tracking technology allows us to identify visual patterns involved in the decision-making. The aim of this pilot study was to evaluate two training models for the recognition and treatment of anaphylaxis by laypeople, based on expert assessment and eye-tracking technology. A cross-sectional quasi-experimental simulation study was made to evaluate the identification and treatment of anaphylaxis. 50 subjects were randomly assigned to four groups: three groups watching different training videos with content supervised by sanitary personnel and one control group who received face-to-face training during paediatric practice. To evaluate the learning, a simulation scenario represented by an anaphylaxis' victim was designed. A device capturing eye movement as well as expert valuation was used to evaluate the performance. The subjects that underwent paediatric face-to-face training achieved better and faster recognition of the anaphylaxis. They also used the adrenaline injector with better precision and less mistakes, and they needed a smaller number of visual fixations to recognise the anaphylaxis and to make the decision to inject epinephrine. Analysing the different video formats, mixed results were obtained. Therefore, they should be tested to evaluate their usability before implementation.

## 1. Introduction

An anaphylactic shock is a time-critical emergency situation. The identification of the anaphylaxis depends on the recognition of its signs and symptoms. This reaction can occur from minutes until hours after exposure to allergens [1].

The optimal management of anaphylaxis requires an intramuscular epinephrine injection using an autoinjector [1, 2]. The intramuscular administration of epinephrine in the thigh is widely recognised as the first line of medical treatment for anaphylaxis. The administration must be carried out immediately once a diagnosis of anaphylaxis is suspected [1–3].

It is estimated that anaphylaxis affects around 0,05–2% of the population, being the most frequent incident among children and adolescents [4]. Kids spent a great part of their time at school; therefore, the training of teachers and caregivers in first aid has been strengthened in later years [5–7].

Another relevant aspect in time-critical emergency has to do with decision-making. Decision-making in emergency is an important responsibility but difficult to study since it is an internal process that occurs rapidly [8]. Eye-tracking technology has been widely used in the fields of marketing and sports [9, 10] to recognise decision-making processes and has recently been introduced into the clinical field [11].

Therefore, the aim of this pilot study was to evaluate two training models (video versus face-to-face) for the recognition and treatment of anaphylaxis by teachers, based on expert assessment and eye-tracking technology.

## 2. Materials and Methods

### 2.1. Sample

A convenience sample consisting of 50 teacher assistants from the Faculty of Education and Sport Sciences of University of Vigo participated in this study. The sample was formed by 26 men and 24 women, with an average age of 24 ± 7 years.

Their participation was voluntary and no one was benefitted or disadvantaged by participating in the study. Participants with previous training or experience of using epinephrine autoinjector were excluded.

All participant signed written informed consent forms, authorising the use of their data. After collection, all data was anonymised. This study followed the ethical principles of the Helsinki Declaration. The Spanish legislation does require approval from an ethical committee for noninvasive simulation studies.

### 2.2. Design

**Figure 1:**
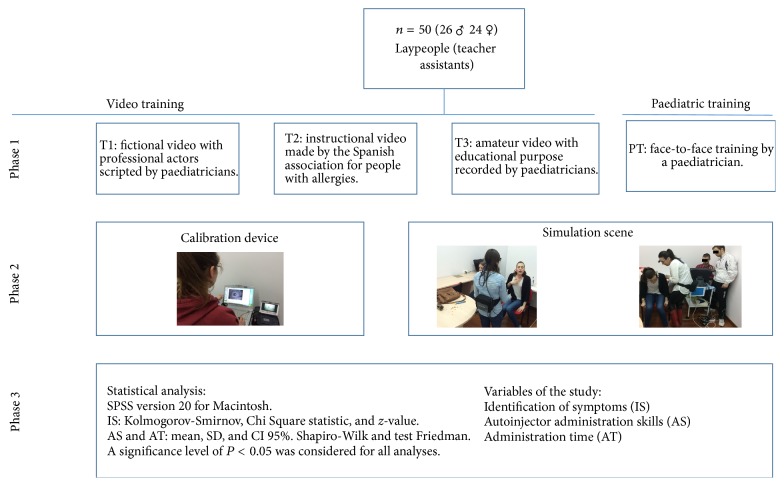
Flowchart.

A controlled simulation study was made to evaluate the identification of signs and symptoms of the anaphylactic reaction and its emergency treatment. For this a cross-sectional quasi-experimental study design was used (Figure 1).

#### 2.2.1. Training in Recognising and Treating Anaphylaxis

The subjects were randomly allocated into four groups, to which we assigned four different training methods. The content of the training was about the recognition of anaphylaxis, as well as its emergency treatment using an epinephrine injector. Three of the groups received their training through videos and the fourth received a face-to-face training by a paediatrician (PT) (see online Supplementary Video in Supplementary Material, available online at https://doi.org/10.1155/2017/9837508). The training time never exceeded ten minutes.

#### 2.2.2. Methodology of the Videos

Training 1 (T1) consisted of a fictional video with professional actors, scripted by paediatricians (https://www.youtube.com/watch?v=v8qI6qL8sv0). Training 2 (T2) was based on an instructional video made by the Spanish association for people with allergies (https://www.youtube.com/watch?v=C9H2Adjkh7c). Training 3 (T3) consisted of an amateur video with educational purpose recorded by paediatricians (https://www.youtube.com/watch?v=8kEJSzGx4_4).

### 2.3. Variables and Evaluation System

To begin with, demographic data of sex and age were recorded. Thereafter the variables to be used for the study were collected: (A) identification of symptoms (IS), (B) autoinjector administration skills (AS), and (C) administration time (AT); see Table 1.

**Figure 2:**
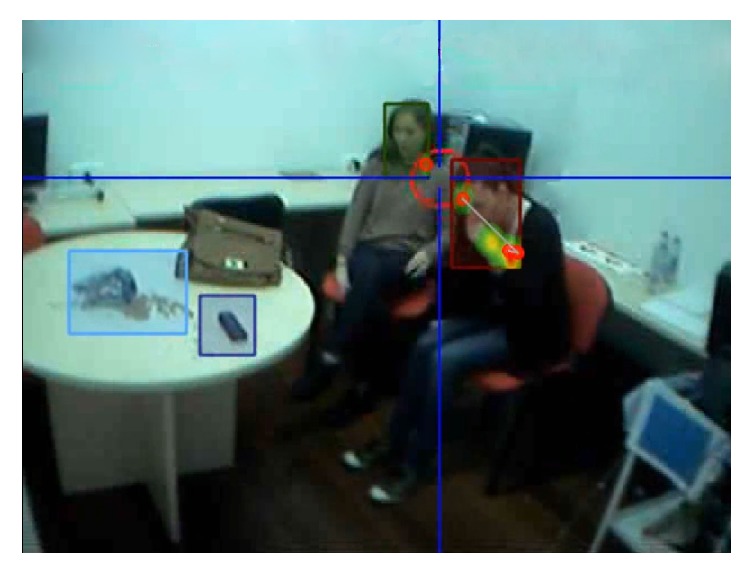
Eye movement analysis.

To evaluate the IS, the eye movement was registered by Mobile Eye from ASL Laboratory (Bedford, USA) (Figure 2). It is composed of two cameras mounted on a pair of light glasses. One to record the scene, and the other one uses the reflection on a lens produced by the cornea and the pupil, to capture were the participants focus their vision. Both signals are registered through its DVCR recording unit and merged by the computer system, producing a compound image of the environment observed together with the participant's visual fixations. The Mobile Eye system was calibrated using the Eye Vision 2.2.5 software, and the videos rendered during the study were analysed using the ASL Result Plus Gaze Map software. Both software packages were run on an ACER ASPIRE 5920G. To evaluate the AS and AT, two expert nurses with experience of out-of-hospital emergencies were responsible for registering the variables using a checklist.

### 2.4. Simulation Scenario

The scenario was designed by experts in simulations and emergencies. The case design was based on clinical criteria for diagnosing anaphylaxis from the World Allergy Organization Guidelines for the Assessment and Management of Anaphylaxis [1]. To assure consistency between the simulations, the case was programed so each event happened in the same moment. Allowing identical synchronisation between all session.

The participant, wearing the eye-tracking device, entered the room were the anaphylactic shock simulation was taking place. Before entering, they were asked to make decisions and act as if it was a real situation.

**Figure 3:**
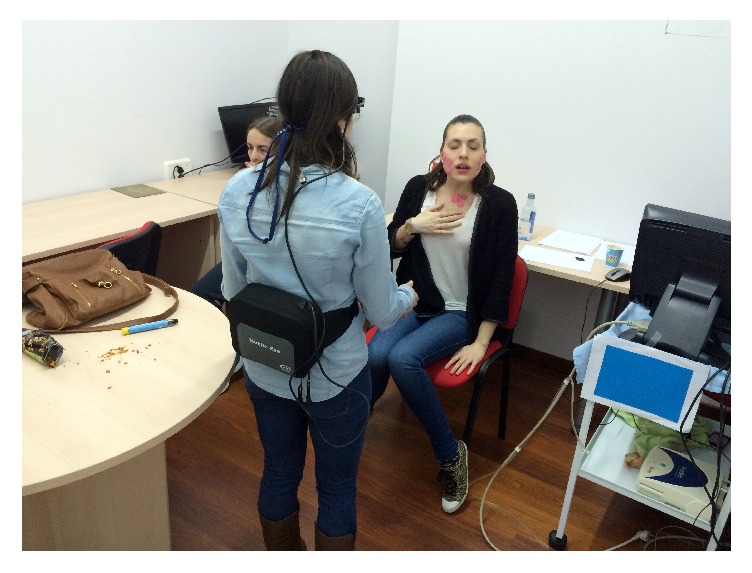
Cutaneous symptomatology.

They encountered two collaborators in the room, each sitting on a chair near a table. One of them, a 27-year-old woman, represented the clinical criteria for diagnosing anaphylaxis [1]: acute appearance of a disease with cutaneous affectation (itching, urticarial, and morbilliform rash) and compromised respiratory function (cough, dyspnea, increased respiratory rate, shortness of breath, and chest tightness). For this purpose, the victim was wearing make-up simulating the cutaneous symptomatology of exposure to an allergen (Figure 3).

The allergen that triggered the anaphylactic shock (roasted corn kernels), a telephone, and a women's purse, simulating the victim's personal belongings were placed on top of the table in front of the simulated victim. The purse contained two epinephrine autoinjector trainers (Jext® Trainer, ALK-Abelló, Hoersholm, Denmark).

The goal for the participants was to identify the person that was suffering an anaphylactic shock and—after calling emergency services—find the epinephrine injector to make the administration.

Two researchers oversaw the whole experiment from one part of the room, evaluating the autoinjector administration skills (AS) and the administration time (AT). None of them gave any information to the participants during the experiment.

The simulation finished when the participants administrated the adrenaline, when they stated that they had finished, or if the participants had not performed any action in 100 seconds.

### 2.5. Statistical Analysis

The statistical analysis was made with SPSS version 20 for Macintosh (version 20.0, Chicago, IL, USA).

For the variables identification of symptom (IS), the normality distribution of the data was checked with the Kolmogorov-Smirnov test completed by the Lilliefors method. To test the homogeneity of distribution among groups (T1, T2, T3, and PT) frequencies per nominal variable were obtained by analysing the differences between the observed and expected frequency, while using the Chi Square statistic (*χ*^2^). We performed a post hoc analysis when the Chi Square test turned out to be significant; this was done in a similar way as proposed by Beasley & Schumacher [12] using the corrected residuals (*z*-value). A level of −1.96 <* z*-value > 1.96 was considered significant. If the degrees of freedom (df) are above 1 (in the opposite case the phi coefficient would be used), the appropriate statistical index to determine the size of the effect is Cramér's *V* (*V*) [13]. For Chi Square analysis, the magnitude of the effect size was measured by calculating Cramér's *V*. According to Cramér [14] if df = 3, *V* = 0.06 to 0.17 describes a small effect, *V* = 0.18 to 0.29 describes a medium effect, and *V* ≥ 0.30 describes great effect. For each of the tests used in this research the significance value stood at the *P* < 0.05 level.

The variables of autoinjector administration skills (AS) and administration time (AT) were described using measures of central tendency (mean) and of dispersion (standard deviation and a 95% confidence interval). Normality was tested using Shapiro-Wilk's test. Friedman's test was used to compare the variables between groups. A significance level of *P* < 0.05 was considered for all analyses.

## 3. Results

### 3.1. Identification of Symptom (IS)

Significant differences for the variables S1 and S3 were found between the groups PT and T3. No other significant results were found (Table 2).

The participants from the PT group made less visual fixations towards the victim's symptoms [S1 (8.7 ± 9.7), S2 (0.6 ± 1), and S3 (0.5 ± 1)], whereas the participants from the T1 [S1 (36.3 ± 53.9), S2 (5.6 ± 9.9), and S3 (4.9 ± 11.5)] and T3 [S1 (34.7 ± 30), S2 (3.2 ± 7.4), and S3 (2.2 ± 3.2)] groups made more visual fixations towards the symptoms.

### 3.2. Autoinjector Administration Skills (AS)

The distribution of nominal variables is presented in Table 3. Total distribution by nominal variable reveals an unequal distribution for each of the groups in autoinjector error (*χ*^2^ = 17.408, *P* < 0.001, *V* = 0.59), thigh localization (*χ*^2^ = 14.949, *P* < 0.01, *V* = 0.55), quality administration (*χ*^2^ = 8.269, *P* < 0.05, *V* = 0.25), and efficient administration (*χ*^2^ = 13.413, *P* < 0.01, *V* = 0.52). For the autoinjector error variable, the *z*-values show that the PT (good: *z*-value = 3.5; bad*: z*-value = −3.5) and the T3 (good: *z-*value = 3.4; bad*: z*-value = −3.4) are away from the average values. Regarding the PT group, a higher probability of correct use of the injector is seen (good: *z*-value = 3.5) and a lower probability of correct use for the T3 group (bad: *z*-value = 3.4). For the T1 group a lower probability of administering the injection in the correct musculature is observed (bad *z*-value = 3.8). Lastly, the PT group administered the medicine significantly more efficiently than the other groups (good: *z*-value = 2.8), with the T1 group having the lowest probability of correct administration (bad: *z*-value = 2.2).

The T1 administered the epinephrine injection in the wrong place most of the times (70%) in comparison with the T2 and PT groups that administrated it in the correct place, 92,3% and 91,7%, respectively.

The T2 group made a quality administration in 38,5% of the cases, compared to the PT group that made a quality administration in 33,2% of the cases. Conversely, the efficient administration was widely performed better by the PT group (75%) in comparison with the T2 (53,8%), T3 (20%), and T1 (10%) group.

### 3.3. Administration Time (AT)

The data of the administration time variables is presented in Table 4. The hand time is significantly different comparing T1 to T3 (*P* = 0.034) as well as PT to T3 (*P* = 0.035). There is also a significant difference in use time between T1 and PT (*P* = 0.034) as well as between PT and T3 (*P* = 0.011). The injection time did not differ significantly between any groups.

The PT group had the best results in the administration time variables. They were the second fastest group to grab the epinephrine autoinjector (HT), fastest one to perform the injection (UT; 36 ± 21.5), and fastest one to prepare the autoinjector for administration. Once the injection was initiated, the PT group let the injector remain inserted for 8.7 ± 4.8 seconds.

## 4. Discussion

Anaphylaxis is an emergency in which a rapid intervention is critical. Recognising an anaphylactic shock can be complex. In our study, participants with very basic training showed differences in the ability to recognise and treat anaphylaxis. Different video training has shown success in teaching techniques related to the emergencies [15, 16].

In our study, traditional face-to-face training provided by expert personnel showed better results than video training, contrary to various studies that have shown better effect using video training in teaching CPR [15, 16].

The participants of the PT group recognised the symptoms of anaphylaxis faster and with fewer visual fixations. The participants of this group (PT) were the second fastest to grab the injector (hand time = 27.5 ± 18.5 seconds) and fastest in administering the epinephrine. They performed the correct treatment in 36 ± 21.5 seconds from the start of the simulation. Johnston et al. [17] found in their study that the average time for anaesthesia residents to diagnose anaphylaxis in an operating room simulation was 7.6 ± 2.4 minutes, and the time to administer the epinephrine was 6.5 ± 2.1 minutes. A greater emphasis needs to be put on the correct and early recognition and treatment of anaphylaxis by healthcare professionals [18].

The manufacturers' recommendation for correct administration of epinephrine is to retain the autoinjector in place for ten seconds from the moment of puncture [19, 20]. The participants in our study maintained the injector during 4.6 ± 3.9 (T1), 7 ± 4.9 (T2), 6.8 ± 4.3 (T3), and 8.7 ± 4.8 (PT) seconds. The PT group was closest to the recommended time. The variability existing between different injectors [21, 22] in the rate of drug administration led us to include efficacy of administration, using a second administration time. The injector used in our study delivers more than 70% of the epinephrine in three seconds [21]; therefore we considered it as efficient if the administration time exceeded this limit. The World Allergy Organization Guidelines for the Assessment and Management of Anaphylaxis [1] do not mention the adequate time that the epinephrine injector should be maintained inserted.

The study by Arga et al. [23] in which they assessed the ability of physicians to use epinephrine injectors before and after receiving theoretical and practical training showed that errors in the use of the injectors may be related to their designs. In our study, incorrect use of the autoinjector was more frequent in the groups receiving video training (T1: 93.5%; T2: 46.2%; and T3: 80%) than in the group receiving face-to-face training, in which no one committed any errors during the use of the autoinjector. Considering our results, usage errors of the autoinjector seem more related to the received training than to the design of the injector. In the study by Grouhi et al. [24] which evaluated the abilities of physicians, nurses, and pharmacists to use the epinephrine autoinjectors, 75% of the participants did not demonstrate the ability to use the device properly. In our study the percentage varied from 100% (T1) incorrect use, to 93.5% (T3), 66.7% (PT), and 61.5% (T2), respectively.

Participants receiving face-to-face training are more likely to perform an effective administration. These participants made fewer visual fixations towards the symptoms before, during, and after the administration than the rest of the groups. This could be because, due to their training, they recognise the signs of the anaphylaxis faster and therefore do not need as many visual fixations. In contrast, the T1 and T3 groups made a higher number of visual fixations before, during, and after (S1, S2, and S3) the administration and therefore performed it worse than the T2 and PT. Understanding the decision-making process in emergency situations can optimize the training of and recommendations for first responders. In this sense, eye-tracking technology allows us to identify the visual variables involved in the decision-making process [9, 10].

To our knowledge, no study of anaphylaxis treatment performance using eye-tracking and expert assessment has been made. We have found that training by a healthcare expert increases the likelihood of proper use and rapid and efficient administration of epinephrine autoinjector. Future researches should be focused on the use of the eye-tracking in other different scenarios. Brockow et al. showed that a standardized 6-h educational intervention improves the knowledge and practical emergency management skills just after the training and after 3 months [7]. Differences between a brief and a standardized training could contribute to structuring better programs, methodology, and contents of anaphylaxis training courses.

## 5. Limitations

As well as other simulation studies, the results should be interpreted with caution in the case of real victims.

The results should not be generalized due to the existing variability in the autoinjectors.

The efficacy of an administration will depend on the type of injector and its characteristics (epinephrine injection rate, puncture depth, and needle length).

## 6. Conclusions

Face-to-face training by a paediatrician improves the ability to recognise anaphylaxis and the probability of correct use of injector. Training videos can be a useful resource but has a great variability in its efficacy.

## Supplementary Material

Eye movement analysis on the training videos and on the simulation scenario.

## Figures and Tables

**Table 1:** Variables of the study.

(A) Identification of symptoms (IS)	(B) Autoinjector administration skills (AS)	(C) Administration time (AT)
Signs and symptoms that indicate presence of anaphylactic shock on cutaneous (pruritus and morbilliform rash) and respiratory (dyspnea and cough) level. Symptoms 1 (S1): number of visual fixations towards the signs and symptoms that the participant makes before using the autoinjector. Symptoms 2 (S2): number of visual fixations towards the signs and symptoms that the participant makes during the administration of the autoinjector. Symptoms 3 (S3): number of visual fixations towards the signs and symptoms that the participant makes after the administration of the autoinjector. Symptoms 1 in percentage (S1%): percentage of visual fixations towards the signs and symptoms that the participant makes before using the autoinjector. Symptoms 2 in percentage (S2%): percentage of visual fixations towards the signs and symptoms that the participant makes during the administration of the autoinjector. Symptoms 3 in percentage (S3%): percentage of visual fixations towards the signs and symptoms that the participant makes after the administration of the autoinjector.	Autoinjector error (AE): incorrect or no use of the injector. Incorrect use was considered as using the injector upside down, no removal of security cap, and/or no firing of the needle during administration. Thigh localization (TL): it was considered as a quality localization if the administration was made in the thigh musculature. Quality administration (AQ): it was considered as a quality administration if (i) the injector was prepared and used in a correct way, (ii) the injection time (IT) was equal to or greater than ten seconds, (iii) the administration was made in the thigh musculature. Efficient administration (AE): an efficient administration was recorded when, even though a quality administration was not performed, it was efficient for the patient. It was considered as an efficient administration when (i) the injector was prepared and used in a correct way, (ii) the injection time (IT) was equal to or greater than three seconds, (iii) the administration was made in the thigh musculature.	Hand time (HT): time (in seconds) from the beginning of the scenario until the participant held the injector in his or her hand. Use time (UT): time (in seconds) from the beginning of the scenario until the injection was made. Injection time (IT): time (in seconds) that the injector remained inserted in the patient's thigh to inject adrenaline.

**Table 2:** Analysis of identification of symptoms (SI).

	*n* = 50												Friedman Test (Sig.)
	T1			T2			T3			PT			
	*n* (10)			*n* (13)			*n* (15)			*n* (12)			
	Mean	SD	CI (95%)	Mean	SD	CI (95%)	Mean	SD	CI (95%)	Mean	SD	CI (95%)	
S1	**36.3**	53.9	−2.2–74.8	**15.1**	21	0.1–30.1	**34.7**	30	15.7–53.8	**8.7**	9.7	2.2–15.2	FP *∗* F3 = 0.011
S2	**5.6**	9.9	−1.5–12.7	**1.5**	3.2	−0.8–3.8	**3.2**	7.4	−1.6–7.9	**0.6**	1	−0.2–1.2	F1 *∗* F2 *∗* F3 *∗* FP > 0.05
S3	**4.9**	11.5	−3.3–13.1	**1.4**	2.3	−0.2–3	**2.2**	3.2	0.1–4.2	**0.5**	1	−0.2–1.2	FP *∗* F3 = 0.046
S1%	**23.5**	24.1	6.3–40.7	**11**	9.1	4.5–17.5	**19.6**	13.4	11–28.1	**15.2**	14.2	5.7–24.7	F1 *∗* F2 *∗* F3 *∗* FP > 0.05
S2%	**3.9**	6.3	−0.6–8.4	**1.8**	3.5	−0.7–4.4	**2.3**	5.7	−1.4–5.9	**1.3**	2.3	−0.2–2.8	F1 *∗* F2 *∗* F3 *∗* FP > 0.05
S3%	**6.5**	14.7	−4–17	**1.3**	1.7	0.1–2.5	**1.6**	2.6	0–3.2	**1.1**	2.6	−0.7–2.9	F1 *∗* F2 *∗* F3 *∗* FP > 0.05

T1, group that received training consisting of a fictional video with professional actors, scripted by paediatricians; T2, group that received training consisting of an instructional video made by the Spanish association for people with allergies; T3, group that received training consisting of an amateur video with educational purpose recorded by paediatricians; PT, group that received face-to-face training by a paediatrician; S1, number of visual fixations towards the signs and symptoms that the participant makes before using the autoinjector; S2, number of visual fixations towards the signs and symptoms that the participant makes during the administration of the autoinjector; S3, number of visual fixations towards the signs and symptoms that the participant makes after the administration of the autoinjector; S1%, percentage of visual fixations towards the signs and symptoms that the participant makes before using the autoinjector; S2%, percentage of visual fixations towards the signs and symptoms that the participant makes during the administration of the autoinjector; S3%, percentage of visual fixations towards the signs and symptoms that the participant makes after the administration of the autoinjector.

**Table 3:** Analysis of autoinjector administration skills (AS).

Variable	*n* = 50												*χ* ^2^	df	*P*	*V*
	T1			T2			T3			PT						
	*n* (10)	%	*z*-value	*n* (13)	%	*z*-value	*n* (15)	%	*z*-value	*n* (12)	%	*z*-value				
Autoinjector error (AE)																
Bad	4	**60 **	−0.3	6	**46,2**	0.2	12	**80**	3.4^*∗*^	0	**0**	−3.5^*∗*^	17.408	3	0.001	0.59
Good	6	**40**	0.3	7	**53,8**	−0.2	3	**20**	−3.4^*∗*^	12	**100**	3.5^*∗*^				
Thigh localization (TL)																
Bad	7	**70**	3.8^*∗*^	1	**7,7**	−1.6	3	**21,4**	−0.3	1	**8,3**	−1.5	14.949	3	0.002	0.55
Good	3	**30**	−3.8^*∗*^	12	**92,3**	1.6	11	**78,6**	0.3	11	**91,7**	1.5				
Quality administration (AQ)																
Bad	10	**100**	1.8	8	**61,5**	−1.9	14	**93,3**	1.6	8	**66,7**	−1.3	8.269	3	0.041	0.41
Good	0	** 0**	−1.8	5	**38,5**	1.9	1	**6,7**	−1.6	4	**33,3**	1.3				
Efficient administration (AE)		** **														
Bad	9	**90**	2.2^*∗*^	6	**46,2**	−1.2	12	**80**	1.9	3	**25**	−2.8^*∗*^	13.413	3	0.004	0.52
Good	1	**10**	−2.2^*∗*^	7	**53,8**	1.2	3	**20**	−1.9	9	**75**	2.8^*∗*^				

*Notes*. *χ*^2^: Chi square; *P*: significance; *V*: Cramér's *V*; OR: odds ratio; df: degrees of freedom; *z*-values indicate if the observed count within a specific cell is significantly larger or smaller than the expected count under the null hypothesis of no association between the variables. ^*∗*^Significant difference: −1.96 < *z*-value > 1.96. T1, group that received training consisting of a fictional video with professional actors, scripted by paediatricians; T2, group that received training consisting of an instructional video made by the Spanish association for people with allergies; T3; group that received training consisting of an amateur video with educational purpose recorded by paediatricians; PT, group that received face-to-face training by a paediatrician.

**Table 4:** Analysis of administration time (AT).

	*n* = 50												Friedman
	T1			T2			T3			PT			
	*n* (10)			*n* (13)			*n* (15)			*n* (12)			
	Mean	SD	CI (95%)	Mean	SD	CI (95%)	Mean	SD	CI (95%)	Mean	SD	CI (95%)	
HT	**26.8**	13.9	15.2–38.3	**39**	33.2	17.9–60.1	**55.6**	39.6	31.7–79.5	**27.5**	18.5	15.8–39.2	F1 *∗* F3 = 0.034
													FP *∗* F3 = 0.035
UT	**59.8**	39.3	26.9–92.6	**58.1**	34.6	36.1–80.1	**73.1**	35.6	51.6–94.6	**36**	21.5	22.3–49.7	F1 *∗* FP = 0.034
													FP *∗* F3 = 0.011
IT	**4.6**	3.9	1.4–7.9	**7**	4.9	3.9–10.1	**6.8**	4.3	4.2–9.4	**8.7**	4.8	5.7–11.7	F1 *∗* F2 *∗* F3 *∗* FP > 0.05

T1, group that received training consisting of a fictional video with professional actors, scripted by paediatricians; T2, group that received training consisting of an instructional video made by the Spanish association for people with allergies; T3, group that received training consisting of an amateur video with educational purpose recorded by paediatricians; PT, group that received face-to face training by a paediatrician; HT, hand time; UT, use time; IT, injection time.
